# Supplementary material for: Establishing a protocol for the compatibilities of closed-system transfer devices with multiple chemotherapy drugs under simulated clinical conditions
Source: PLoS One. 2021 Sep 28;16(9):e0257873. doi: 10.1371/journal.pone.0257873 (PMC8478200; doi:10.1371/journal.pone.0257873)
Supplement: S1 Table — (DOCX) [file pone.0257873.s001.docx]

**S1 Table. Drug selection.**

| **Drugs** | **Selection criteria** | **Polarity: topological Polar Surface Area (Å²)^b^** | **pH^c^** |
| --- | --- | --- | --- |
| Busulfan | FDA warning^a^, Solvent contains DMA | 104 | 3.4-3.9 |
| Etoposide | Ethanol, polysorbate 80 | 161 | 3.0-4.0 |
| Paclitaxel | Cremophor, Ethanol | 221 | 3.0-7.0 |
| Melphalan | Ethanol (96%) | 66.6 | 3.0-7.0 |
| Cisplatin | Most commonly used anticancer drug | 2 | 3.5-4.5 |
| Cyclophosphamide | Most commonly used alkylating agent | 41.6 | 6.87 |
| Fluorouracil | Most commonly used antimetabolite | 58.2 | 8.6-9.4 |
| Irinotecan | Most commonly used topoisomerase I | 113 | 3.0-4.0 |
| Doxorubicin | Most commonly used topoisomerase II | 206 | 3.8-6.5 |
| Vinorelbine | Most commonly used mitosis inhibitor | 134 | 3.5 |

^a^ The US Food and Drug Administration warned against the use of busulfan with CSTD.

^b^ Information obtained by PubChem. https://pubchem.ncbi.nlm.nih.gov/

^c^ Information obtained by 18ed Handbook on Injectable Drugs.
