# Supplementary material for: Establishing a protocol for the compatibilities of closed-system transfer devices with multiple chemotherapy drugs under simulated clinical conditions
Source: PLoS One. 2021 Sep 28;16(9):e0257873. doi: 10.1371/journal.pone.0257873 (PMC8478200; doi:10.1371/journal.pone.0257873)
Supplement: S2 Table — (DOCX) [file pone.0257873.s002.docx]

**S2 Table.** **Drug preparations.**

| **Drugs** | **Drug strength**  **(mg:mL)** | **Diluent** | **Final dilution (mg/mL)** |
| --- | --- | --- | --- |
| Busulfan | 60:10 | NS | 0.6 |
| Etoposide | 1000:5 | NS | 0.4 and 12 |
| Paclitaxel | 30:5 | NS | 1.2 |
| Melphalan | 50:10 | NS | 0.45 |
| Cisplatin | 50:50 | NS | 0.9 |
| Cyclophosphamide | 500:25 | NS | 4 |
| Fluorouracil | 1000:20 | NS | 45 |
| Irinotecan | 100:5 | D5W | 2.8 |
| Doxorubicin | 10:5 | NS | 0.18 |
| Vinorelbine | 10:1 | NS | 2 |

NS, normal saline 0.9% sodium chloride; D5W, 5% dextrose in water solution; DMA, N, N-dimethylacetamide
