# Supplementary material for: Establishing a protocol for the compatibilities of closed-system transfer devices with multiple chemotherapy drugs under simulated clinical conditions
Source: PLoS One. 2021 Sep 28;16(9):e0257873. doi: 10.1371/journal.pone.0257873 (PMC8478200; doi:10.1371/journal.pone.0257873)
Supplement: S3 Table — (DOCX) [file pone.0257873.s003.docx]

**S3 Table. HPLC conditions for analysis of antineoplastic drugs.**

| **Drugs** | **Mobile Phases** | **Flow rate (mL/min)** | **Retention time (min)** | **Injection Volume (uL)** | **wavelength (nm)** | **Dilution factor** |
| --- | --- | --- | --- | --- | --- | --- |
| Etoposide  (low & high) | 0.1% FA/ACN  (85:15 and 50:50 v/v) | 1.2 | 10 | 5 | 285 | 50 |
| Paclitaxel | Water/ACN (40:60 v/v) | 1 | 3.5 | 5 | 275 | 1 |
| Melphalan | 0.1% FA/ACN  (70:30 v/v) | 1.2 | 3 | 5 | 275 | 1 |
| Cisplatin | Water/Methanol  (40:60 v/v) | 0.5 | 5 | 5 | 204 | 1 |
| Cyclophosphamide | 0.1% FA/ACN  (85:15 and 50:50 v/v) | 1.2 | 9 | 5 | 285 | 1 |
| Fluorouracil | 0.1% FA/ACN  (85:15 v/v) | 0.5 | 3.5 | 5 | 300 | 1 |
| Irinotecan | 0.1% FA/ACN  (85:15 and 50:50 v/v) | 1.2 | 8 | 5 | 254 | 50 |
| Doxorubicin | 0.1% FA/CAN  (85:15 and 50:50 v/v) | 1.2 | 8 | 5 | 254 | 5 |
| Vinorelbine | 0.1% FA/ACN  (85:15 and 50:50 v/v) | 1.2 | 8 | 5 | 330 | 1 |

FA, formic acid; ACN, acetonitrile
