# Supplementary material for: Establishing a protocol for the compatibilities of closed-system transfer devices with multiple chemotherapy drugs under simulated clinical conditions
Source: PLoS One. 2021 Sep 28;16(9):e0257873. doi: 10.1371/journal.pone.0257873 (PMC8478200; doi:10.1371/journal.pone.0257873)
Supplement: S4 Table — (DOCX) [file pone.0257873.s004.docx]

**S4 Table. Comparison of the results of two CSTD compatibility studies and the present study.**

|  | **Our Study** | **Report no. 1** | **Report no. 2** |
| --- | --- | --- | --- |
| Test devices | Vial access clips  Vial access spikes  Administration set | Closed male luer  Vial spikes  Administration sets | Vial adaptors  Syringe Units  Secondary sets  Y-set |
| Commercial drug used | Yes | Not specified | Yes |
| Drug selection | **Inactive ingredients:**  Busulfan, etoposide, paclitaxel, melphalan  **Therapeutic class:** cisplatin, cyclophosphamide, fluorouracil, irinotecan, doxorubicin, vinorelbine, | **Inactive ingredients:**  Etoposide, paclitaxel  **Therapeutic class:**  Bevacizumab, cetuximab, cisplatin, cyclophosphamide, doxorubicin, fluorouracil, trastuzumab, methotrexate, vincristine | **Inactive ingredients:**  Busulfan (0.54 mg/mL), paclitaxel (1.2 mg/mL), bendamustine (0.75 mg/mL)  **Therapeutic class:**  Etoposide (0.4 mg/mL) |
| Drug compounding dilution | Highest therapeutic concentrations | Three times therapeutic values | Diluted with double distilled water (DDW solution) to therapeutic concentrations according to USP guideline |
| Maximum vial storage time | 8 h at 25℃ | Closed male luer: 120 days of refrigeration and 7 days at room temperature  Vial spikes: 30 days of refrigeration and 7 days at room temperature | 24 h at 25℃ |
| Maximum simulated infusion time | 24 h at 25℃ | 24 h at room temperature | 24 h at 25℃ |
| Analysis parameters | Potency maintenance  Plasticizer migration  Device functionality | Drug stability test  Plastic migration test  Functional integrity test | Compare with standard PP syringe  Drug stability  Device functionality |
