# Supplementary material for: Establishing a protocol for the compatibilities of closed-system transfer devices with multiple chemotherapy drugs under simulated clinical conditions
Source: PLoS One. 2021 Sep 28;16(9):e0257873. doi: 10.1371/journal.pone.0257873 (PMC8478200; doi:10.1371/journal.pone.0257873)
Supplement: Attachment — Submitted filename: Response to reviewers.docx [file pone.0257873.s005.docx]

PONE-D-20-34542

Response to Reviewers

Dear Professor Sailor,

We have modified our manuscript according to POLS ONE’s style requirements, and the competing interest statement in our cover letter have been amended as requested.

Please review our responses below regarding the reviewer’s comments:

**Reviewer #1:**

INTRODUCTION
1. ONB - spell in full first before being used as an abbreviation.

ONB is the product code for the product classification of Closed Antineoplastic and Hazardous Drug Reconstitution and Transfer System by the FDA. It is not an abbreviation.

2. With reference to "manufacturer's claim: stated in Line 46, it is suggested to give a brief detail of such claims, relevant to this study.

We have made modifications, please refer to Line 48 in the revised manuscript.
MATERIALS AND METHODS
1. Where application, give the references for the method adopted in the work.

We do not have further reference material to provide.

2. Line 120, why half of the total dosage required to prepare one intravenous infusion bag was used? A brief clarification is required.

It has been clarified, please refer to Line 138-139 in the manuscript.

3. Line 176: It is suggested to give the source for the specifications.

We have provided an additional reference for the source. Please see reference 21.

4. Lines 182, 186, 195, 202 and 205 (or where applicable): It is suggested to enlist the materials used under the heading of Materials. They should not be mingled with the methods.

The listed materials have been moved to appropriate sections.
5. There is a need for a brief description of "simulated clinical conditions" in Materials and Methods.

This has been addressed please refer to Line 127.

CONCLUSION
1. There is a need to put the heading of the conclusion.

The heading has been added.
2. A sentence on the "maintenance of efficacy" should be a part of conclusion.

This has been addressed in the conclusion. Please see the revised version.

**Reviewer #2:**

Each test was performed in triplicate (page 5, line 102), but appeared to have been done by the same pharmacist (page 6, line 113). They should be done by different pharmacists, preferably on different days. This is the limitation of this study.

We had included a “study limitation” section within the discussion, please refer to Line 361.

Please do not hesitate to contact me should there be further clarification needed regarding our manuscript. Thank you for your time.

Best regards,

Shao-Chin Chiang, PharmD, RPh

Department of Pharmacy
